# Correction: A chip-scale second-harmonic source via self-injection-locked all-optical poling

**DOI:** 10.1038/s41377-025-02002-w

**Published:** 2025-10-13

**Authors:** Marco Clementi, Edgars Nitiss, Junqiu Liu, Elena Durán-Valdeiglesias, Sofiane Belahsene, Hélène Debrégeas, Tobias J. Kippenberg, Camille-Sophie Brès

**Affiliations:** 1 Photonic Systems Laboratory (PHOSL), École Polytechnique Fédérale de Lausanne, 1015 Lausanne, Switzerland; 2 Laboratory of Photonics and Quantum Measurements (LPQM), École Polytechnique Fédérale de Lausanne, 1015 Lausanne, Switzerland; 3 Almae Technologies, Route de Nozay, 91460 Marcoussis, France

**Keywords:** Integrated optics, Nonlinear optics, Diode lasers, Semiconductor lasers

Correction to: *Light: Science & Applications* 10.1038/s41377-023-01329-6, published online 08 December 2023

Following publication of the original article, an error was identified in **Figure 3b** of the published version: the y-axis label is incorrectly listed as “**mV**” (millivolt) instead of the correct unit “**mW**” (milliwatt). The correct figure 3 is shown below:

Incorrect Figure 3
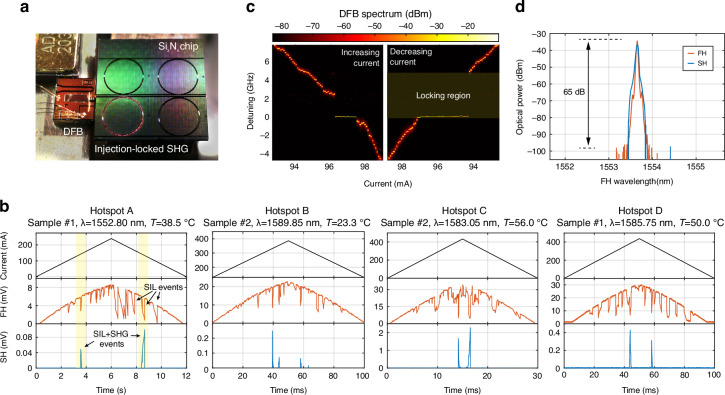


Correct Figure 3
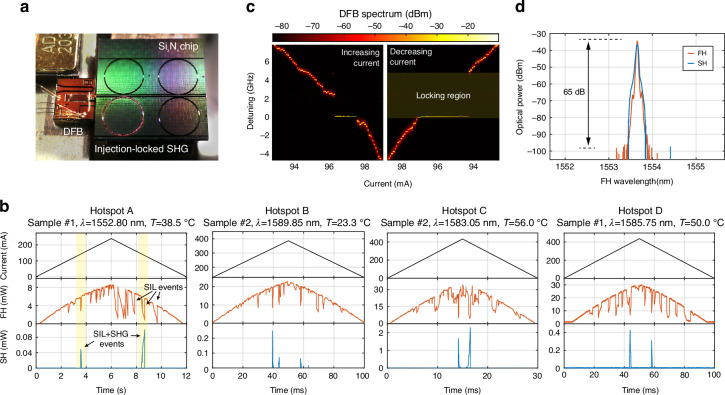


The original paper has been updated.

