# Supplementary material for: A chip-scale second-harmonic source via self-injection-locked all-optical poling
Source: Light Sci Appl. 2023 Dec 8;12:296. doi: 10.1038/s41377-023-01329-6 (PMC10703906; doi:10.1038/s41377-023-01329-6)
Supplement: Supplementary file 1 — Supplementary Information [file 41377_2023_1329_MOESM1_ESM.pdf]

Supplementary Information for:
A chip-scale second-harmonic source via self-injection-locked all-optical poling

Marco Clementi,[1,*] Edgars Nitiss,[1] Junqiu Liu,[2] Elena Durán-Valdeiglesias,[3] Sofiane Belahsene,[3] Hélène Debrégeas,[3] Tobias J. Kippenberg,[2] and Camille-Sophie Brès[1,†]

[1] *Photonic Systems Laboratory (PHOSL), École Polytechnique Fédérale de Lausanne, 1015 Lausanne, Switzerland*
[2] *Laboratory of Photonics and Quantum Measurements (LPQM), École Polytechnique Fédérale de Lausanne, 1015 Lausanne, Switzerland*
[3] *Almae Technologies, Route de Nozay, 91460 Marcoussis, France*

Supplementary Note 1. Experimental setup

A full schematic of the experimental setup, in various operating configurations, is shown in Supplementary Figure 1. The configuration used for linear spectroscopy and for mapping of the AOP is shown in panel 1a. Here, a tunable laser operating at telecom wavelength is amplified and coupled to the Si_3N_4 chip using a lensed fiber. The residual pump and the generated SH are collected at the output using an aspheric lens, separated by a dichroic mirror and detected at photodetectors. The backreflected signal is also recorded using a circulator, in order to verify the presence of resonant backscattering from the microring. The output data from the three photodetectors is recorded by an oscilloscope. An electro-optic phase modulator driven at high frequency (1 GHz) was used to calibrate the measurement via sideband spectroscopy[1]. The power inside the waveguide and the reflectivity were estimated by accounting for the loss at collection and in-coupling. The configuration used for SIL-SHG is shown in panel 1b. Here, the table-top amplified source is replaced by a DFB, whose current and temperature are controlled by external electronics. The diode current can be also swept using a function generator, in order to scan the operating wavelength. The output of the setup is collected using either achromatic free-space optics or a lensed fiber (not shown), and routed to an optical spectrum analyzer or demultiplexed and photodetected to visualize the FH and SH time traces. An isolator is used on the demultiplexed FH channel to prevent any backreflection that could interfere with the SIL process. The detection stage (shaded gray area) is replaced by the apparatuses shown in panels 1c and 1d for the measurements of optical heterodyne and frequency noise, respectively. Optical heterodyne (panel 1c) is implemented by mixing the output FH field with the light from a reference source at telecom wavelength (local oscillator) at a 50:50 fiber beamsplitter. The output signal is retrieved by a fast photodetector and acquired by an electrical spectrum analyzer. For the frequency noise measurement, we implemented two identical frequency discriminators (panel 1d), fed with the demultiplexed FH and SH outputs, respectively. Such systems consist in an unbalanced Mach-Zehnder interferometer, which is used to map phase fluctuations into amplitude fluctuations. The first output is fed to a fast photodiode, while the second one is used to monitor the output power. The relative phase between the arms controlled by a fiber phase shifter, which is driven by a triangular wave in order to average (scramble) the response of the discriminators. The output traces are recorded simultaneously by an oscilloscope or an electrical spectrum analyzer. In the former case, the power spectrum is retrieved by a numerical Fourier transform, while the measurement is calibrated on the peak-to-peak response amplitude, assessed after low-pass filtering.

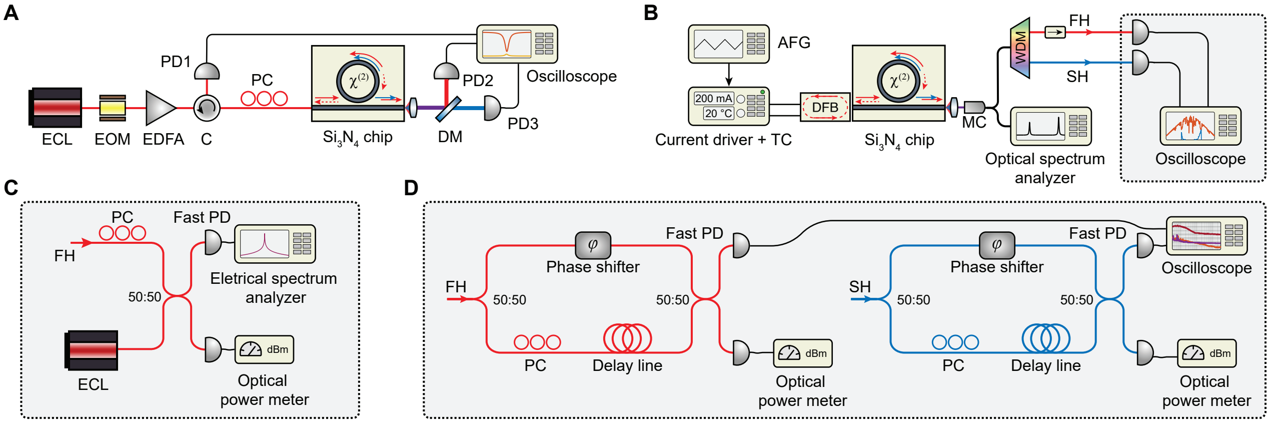

Supplementary Figure 1. Schematic of the experimental setup. **a.** Configuration used for linear spectroscopy and AOP mapping. ECL: external cavity laser, EOM: electro-optic modulator, EDFA: erbium-doped fiber amplifier, C: circulator, PD: photodetector, PC: polarization controller, DM: dichroic mirror. **b.** Configuration used for probing of the SIL-SHG source. AFG: arbitrary function generator, TC: temperature controller, WDM: wavelength division multiplexer, MC: mirror collimator. The gray shaded area is replaced by the apparatuses shown in panels **c.** for heterodyne detection and **d.** for frequency noise measurements, respectively.

Supplementary Note 2. Additional all-optical poling maps

We hereby report, for completeness, the all-optical poling maps for all the 3 samples involved in this study. The maps are shown in Supplementary Figure 2. Panels 2a and 2c are also present in the main text. The FH-SH1 trends are marked by white dashed lines.

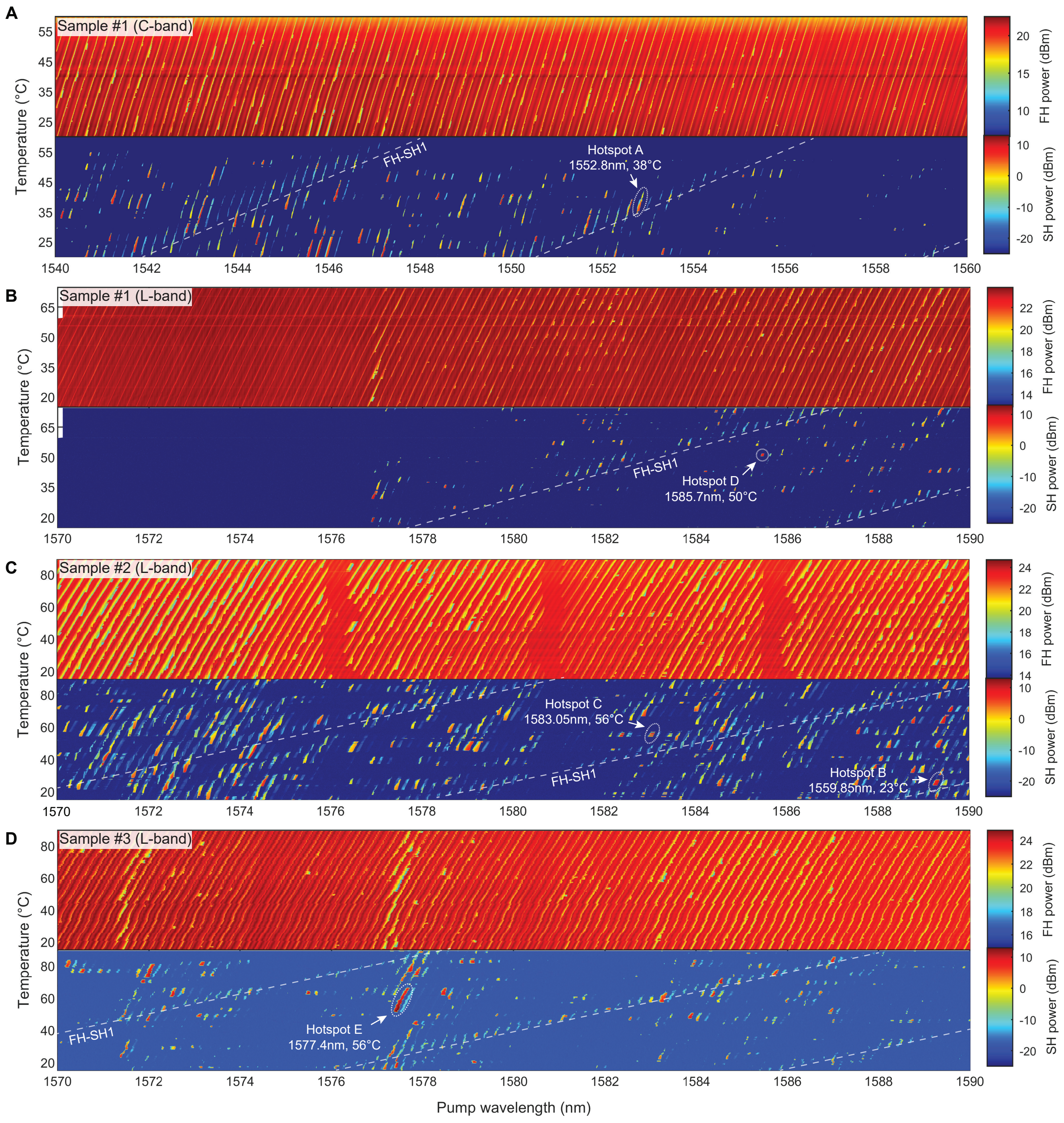

Supplementary Figure 2. All-optical poling maps for the samples studied in this work.

Supplementary Note 3. Estimate of the photoinduced $\chi^{(2)}$

In order to provide an estimate of the second-order nonlinear susceptibility, photoinduced through the coherent photogalvanic effect, we follow a methodology analogous to the one described in Ref.[2]. We first define the nonlinear coupling coefficient:

$$\hbar g \approx \epsilon_0 \frac{3\chi^{(2)}}{4\sqrt{2}} \int (u_{\mathrm{FH}}^*)^2 u_{\mathrm{SH}}\, dV \tag{1}$$

where the approximation entails a constant (effective) value of $\chi^{(2)}$ along the whole round-trip. Here $\hbar$ is the reduced Planck constant, ϵ_0 is the vacuum permittivity and $u_{\mathrm{FH(SH)}}$ is the TE component of the FH (SH) field profile, normalized such that:

$$\int \epsilon_0 n^2 |u_{\mathrm{FH(SH)}}|^2\, dV = \hbar\omega_{\mathrm{FH(SH)}} \tag{2}$$

where n is the refractive index and $\omega_{\mathrm{FH(SH)}}$ is the FH (SH) resonance frequency, expressed in $\mathrm{rad\,s^{-1}}$. With these definitions, the peak conversion efficiency at resonance can be expressed as:

$$\mathrm{CE} = \frac{P_{\mathrm{SH}}}{P_{\mathrm{FH}}^2} = \frac{16g^2}{\hbar\omega_{\mathrm{FH}}} \frac{\eta_{\mathrm{FH}}^2}{\kappa_{\mathrm{FH}}^2} \frac{\eta_{\mathrm{SH}}}{\kappa_{\mathrm{SH}}} \tag{3}$$

where $\kappa_{\mathrm{FH(SH)}}$ is the resonance linewidth (full width at half maximum) at the FH (SH) frequency expressed in $\mathrm{rad\,s^{-1}}$ and $\eta_{\mathrm{FH(SH)}} = \kappa_{0,\mathrm{FH(SH)}}/\kappa_{\mathrm{FH(SH)}}$ is the coupling efficiency, being $\kappa_{0,\mathrm{FH(SH)}}$ the intrinsic linewidth. From comparison between Eqs. (1) and (3), one can explicitly derive the value of $\chi^{(2)}$. In our case, the integral in Eq. (1) was calculated through numerical simulations, the coupling efficiency and linewidth at FH were assessed experimentally through transmission measurements, the coupling efficiency at SH was estimated via finite-difference time domain simulations and the value of κ_{SH} was inferred assuming a propagation loss $\alpha_{\mathrm{SH}} = 4\,\mathrm{dB\,m^{-1}}$.

Supplementary Note 4. Additional frequency noise traces

We hereby report two additional frequency noise traces associated with SIL at two of the hotspots investigated. The traces, shown in Supplementary Figure 3, refer to the FH field only, and display a reduction of the white noise plateau up to almost 4 orders of magnitude (39 dB), with a lowest value (panel 3b) as small as 13 Hz, corresponding to an intrinsic linewidth of 41 Hz. While both of measurements were acquired during SIL-SHG, only the FH traces are available here due to the lower sensitivity at the SH wavelength, which was affected by the lower collected power at the SH.

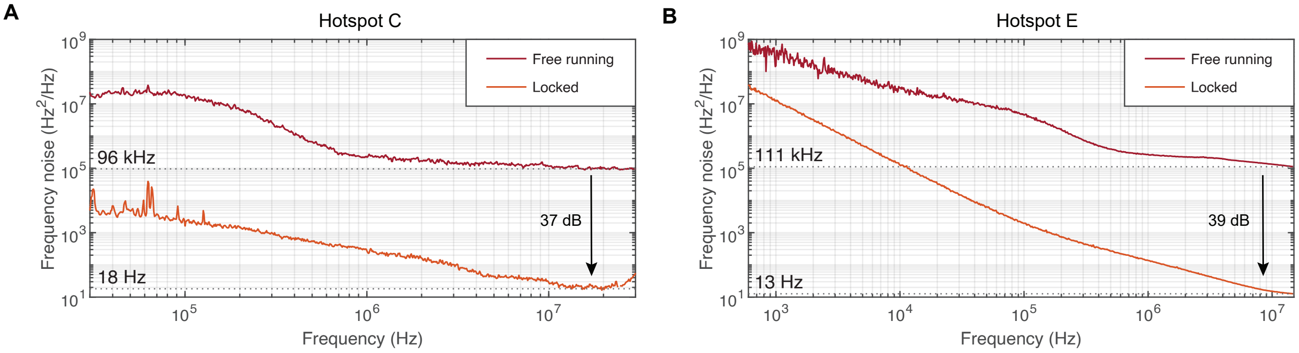

Supplementary Figure 3. Additional frequency noise traces (FH only).

* marco.clementi@epfl.ch

† camille.bres@epfl.ch

[1] J. Li, H. Lee, K. Y. Yang, and K. J. Vahala, Optics Express **20**, 26337 (2012).
[2] X. Guo, C.-L. Zou, and H. X. Tang, Optica **3**, 1126 (2016).
